# Reducing uncertainties in energy dissipation measurements in atomic force spectroscopy of molecular networks and cell-adhesion studies

**DOI:** 10.1038/s41598-018-26979-0

**Published:** 2018-06-20

**Authors:** Soma Biswas, Samuel Leitao, Quentin Theillaud, Blake W. Erickson, Georg E. Fantner

**Affiliations:** Laboratory for Bio- and Nano-Instrumentation, École Polytechnique Fédérale de Lausanne, Batiment BM 3109 Station 17, 1015 Lausanne, Switzerland

## Abstract

Atomic force microscope (AFM) based single molecule force spectroscopy (SMFS) is a valuable tool in biophysics to investigate the ligand-receptor interactions, cell adhesion and cell mechanics. However, the force spectroscopy data analysis needs to be done carefully to extract the required quantitative parameters correctly. Especially the large number of molecules, commonly involved in complex networks formation; leads to very complicated force spectroscopy curves. One therefore, generally characterizes the total dissipated energy over a whole pulling cycle, as it is difficult to decompose the complex force curves into individual single molecule events. However, calculating the energy dissipation directly from the transformed force spectroscopy curves can lead to a significant over-estimation of the dissipated energy during a pulling experiment. The over-estimation of dissipated energy arises from the finite stiffness of the cantilever used for AFM based SMFS. Although this error can be significant, it is generally not compensated for. This can lead to significant misinterpretation of the energy dissipation (up to the order of 30%). In this paper, we show how in complex SMFS the excess dissipated energy caused by the stiffness of the cantilever can be identified and corrected using a high throughput algorithm. This algorithm is then applied to experimental results from molecular networks and cell-adhesion measurements to quantify the improvement in the estimation of the total energy dissipation.

## Introduction

**Figure 1:**
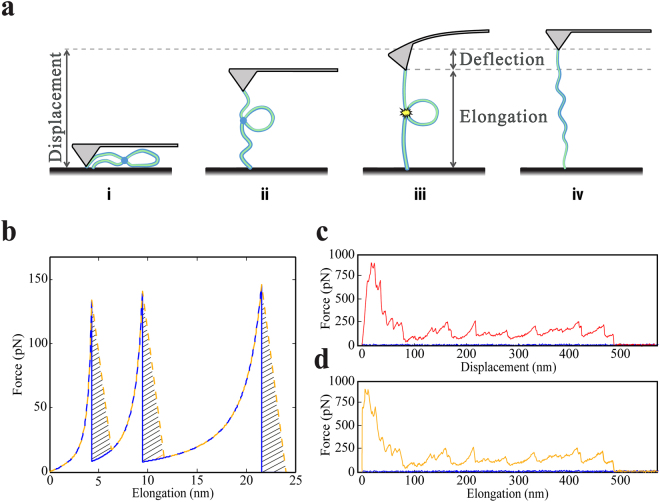
Source of overestimation of the dissipated energy calculated from a complex pulling curve. (**a**) Shows a schematic representation of AFM pulling experiment explaining the displacement (amount of piezo motion), elongation (actual tip-sample distance) and deflection of the cantilever. (**b**) Shows a simulated pulling curve with three peaks using WLC model. The dark blue continuous line represents the ideal WLC curve and the orange dashed line represents the real case scenario (considering the finite stiffness of the cantilever). The force decreases gradually over a distance because of the stiffness of the cantilever after a rupture event occurs. This results in excess energy estimation from SMFS data analysis. The shaded regions marked with dark lines for all the three peaks indicate the areas that contribute to the overestimation in the calculation of energy dissipation. (**c**) Shows a complex pulling curve as generated by the AFM processor without further corrections, where the application of our algorithm is relevant. This curve was taken on Osteopontin deposited on mica surface in presence of Ca-ions in the buffer. (**d**) Shows the same pulling curve as in (**c**) after slanting correction.

Atomic force microscope (AFM) based single molecule force spectroscopy (SMFS), also referred to as “molecular pulling”, has been extensively used to study inter- and intra-molecular interactions and mechanical properties of various biological and synthetic macromolecules. These interactions are involved in many biological processes such as cell surface interaction and adhesion mechanisms^1,2^, protein folding and unfolding^3,4^, small force actuation in DNA and RNA molecules^5,6^, ligand-receptor interactions (such as protein-protein and DNA-protein interaction or antibody-antigen linking)^7–10^ and breaking of sacrificial bonds within the non-collagenous proteins of bone)^11,12^. Recent studies of bacteria-surface interactions using AFM force spectroscopy has demonstrated the potential of this technique in the field of cell adhesion^1,2,13–17^. Characteristic force-distance curves are often observed when individual proteins or network of proteins or DNA are stretched with an AFM tip. Commonly used models for analyzing the force-distance curves of single molecules are the freely jointed chain (FJC) model^18^, the worm-like chain (WLC) model^19,20^ and their modifications^21^. These models can provide valuable information about mechanical properties and structural variants of single molecules. However, the large number of molecules, commonly involved in complex networks formation or cell-adhesion, leads to difficulties in force spectra analysis. For such complex networks it is difficult to decompose the complex force curves into individual single molecule events. One therefore often characterizes the total dissipated energy over a whole pulling cycle instead of the contributions of each individual molecule^11,12,22–24^. For this the area under the force displacement curve is integrated. However, the finite stiffness of the cantilever used for AFM based SMFS causes an overestimation of the calculated dissipated energy value in cases where there are discontinuities such as bond ruptures. At a bond rupture the cantilever performs an uncontrolled snap-back motion, resulting in a region of uncertainty in which no data is present about the true force profile on the molecule (see Fig. 1b). Although this error can be significant, it is generally not compensated for which makes the interpretation of the energy dissipation values sometimes challenging.

Here we present a high throughput algorithm that identifies bond rupture events in complex SMFS curves, and calculates the dissipated energies by interpolating the regions of uncertainty thereby reducing the overestimation of dissipated energy values. In this paper, we show both simulated and experimental results, where the excess dissipated energy caused by the stiffness of the cantilever has been identified and also corrected using the algorithm.

To check the effectiveness of the MATLAB algorithm in case of experimental data, we have studied the mechanical properties of thin layers of human Osteopontin (one of the most abundant non-collagenous proteins in bone) using SMFS. It has already been shown that human Osteopontin can form networks and dissipate large amounts of energy through the breaking of sacrificial bonds and stretching the hidden length without the need for folded domains within the protein^22^. In many natural materials (including bone and nacre), this sacrificial bonds and hidden length mechanism acts as a toughening mechanism, which increases the energy needed to break the material^12,25^. By processing the same experimental data with the new algorithm we find that the conventional way of integrating force curves over estimates the energy dissipation by up to 22% (considering the histogram of the Ca buffer data shown later).

## Results

### Source of overestimation of the dissipated energy

Many molecules are generally involved in the formation of a network in natural materials, resulting in a complex shape of the pulling curve (see Fig. 1c). The energy dissipation during a pulling experiment can be calculated by integrating the area between the zero force level and the contour of the force spectroscopy curve.

For pulling experiments, generally, softer cantilevers (spring constant (k) ≈ 0.01–0.05 N/m) are used to increase the force sensitivity. This however means that the amount the cantilever deflects when pulling on the molecule is significant. Upon breakage of a sacrificial bond, hidden length is released and the force applied on the cantilever is reduced, resulting in a reduced deflection, e.g. a “snap-back”. During the snap-back, the cantilever motion is determined primarily by hydrodynamic forces, and no reliable mechanical data is collected about the molecules under investigation. In the force displacement curve this manifests as a “dead zone” (shaded regions marked with dark lines in Fig. 1b). In this paper, displacement refers to the amount of piezo motion (as shown in Fig. 1a). If the total energy dissipation is calculated simply by integrating the area under the force displacement curve, the energy dissipated by the molecular network will be overestimated by the area of these shaded regions shown in Fig. 1b. Depending on the arrangement of the molecules in the network, this can be a substantial fraction of the total dissipated energy. For achieving the correct dissipated energy value calculated from the force spectroscopy curves, it is, therefore, important to eliminate this excess energy, which is not related to the stretching of the biomolecule. In the following section we derive the overestimation of the energy dissipation by simulating an idealized SMFS experiment.

**Figure 2:**
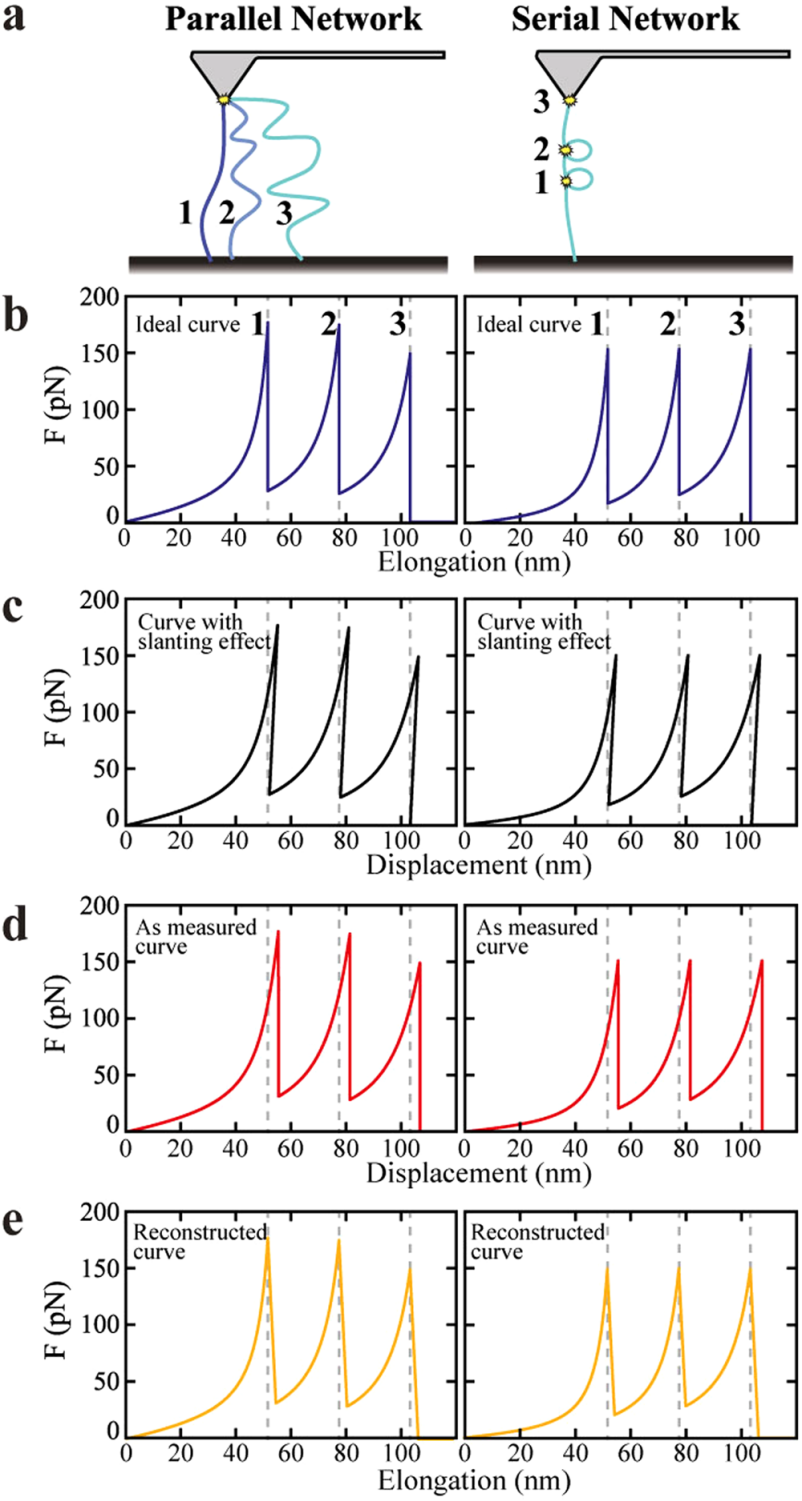
Difference between the ideal (force vs. elongation felt by the molecule) and the real SMFS curves (including the effect of the finite cantilever stiffness). We have simulated a pulling curve with three rupture events using the worm-like chain (WLC) model, for four different conditions implemented in MATLAB. The rupture events were simulated by changes in the contour length (60 nm, 90 nm and 120 nm) of the molecule when the bond rupture force was reached. The values of the bond rupture force and the persistent length used in our model were 150 nN and 0.4 nm respectively. (**a**) Shows the schematic of parallel network of molecules (left hand side) and serial network of sacrificial bonds within the molecule (right hand side) between the tip of the cantilever and the surface. (**b**) Shows the ideal case scenario (force vs. elongation felt by the molecule). (**c**) Shows the force vs. displacement curve taking into account the slanting effect due to the deflection of the cantilever. This curve was generated by implementing a slanting transformation using a slanting factor from the cantilever spring constant (−1/k, where k is the spring constant of the cantilever). It should be noted that this is not a physically measurable curve, but represents cause of the uncertainty in the area right after the snap-off. (**d**) Shows the **“**as measured**”** scenario by replacing the slanted lines in (**c**) with straight lines. (**e**) Shows the reconstructed force vs. elongation curve.

Figure 2a shows the schematic of parallel network of molecules (left hand side) and serial network of sacrificial bonds within the molecule (right hand side) between the tip of the cantilever and the surface. In our model, the parallel network is constituted by considering three molecules in parallel connected to the cantilever with identical bond strength, but different lengths as shown in Fig. 2a (left hand side). The pulling force is distributed over all the three molecules, as they are loaded in parallel. If the distributed force on one molecule exceeds the rupture force of that molecule, it will be detached from the surface. In this case, each molecule breaks at the same distributed force but this force occurs at different elongations. The distance between the neighboring peaks in the pulling curve (Fig. 2b, left hand side) is constant, as the length increase between neighboring molecules was chosen constant for simplicity.

The serial network is constituted by considering three sacrificial bonds of equal strengths within the structure of the molecule. Hidden length is shielded from the pulling force. When the molecule is pulled, an equal amount of force is exerted on all the sacrificial bonds. The sacrificial bonds will break in the order of their lowest bond strength. In this case, the hidden length, which is set free after each rupture, is the distance between the two consecutive binding sites on the molecule. For simplicity, we chose the bond between the molecule and the tip to have the same strength as the sacrificial bond. Thermal fluctuations have not been included into the simulations.

Figure 2b shows the force vs. elongation (elongation refers to the actual tip-sample distance in this paper) as shown in Fig. 1a plot of two hypothetical pulling experiments, one where the hidden length is due to parallel connections of molecules between tip and surface (as is often the case in molecular networks) and one where the hidden length is due to a series of sacrificial bonds within one serial chain (as would be the case for unfolding of proteins)^11^. When pulling on these molecular configurations by AFM with a cantilever with finite stiffness, the force vs. displacement curve differs from the force vs. elongation plot due to the deflection of the cantilever as described above. Mathematically this difference can be described by a slanting transformation of the force vs. elongation curve, resulting in the curves shown in Fig. 2c (this is not a physically measurable curve and the “back slanting” never occurs in reality. This is a simulated curve for illustration purpose only). In this paper, slanting transformation refers to the conversion of displacement (amount of piezo motion) to elongation (actual tip-sample distance) by correcting (subtracting the cantilever deflection from the displacement) for the deflection of the cantilever. The previously vertical drops in the force vs. elongation plot due to breakage of the sacrificial bond now are slanted. However, during the actual measurement, the AFM cantilever will snap back quickly, without any change in the displacement as measured by the sensor governing the displacement of the Z-piezo. In the “as measured” force curve, this manifests in a vertical drop in the force vs. displacement curve as shown in Fig. 2d. This curve would be the raw data as measured with AFM SMFS. Typically, this data is then processed to extract the force vs. elongation of the molecule using standard processing software (such as Bruker NanoScope software) using a slanting transformation in the other direction. The result is the reconstructed force vs. elongation curve shown in Fig. 2e. By comparing Fig. 2b with Fig. 2e it is apparent that the area under the force displacement curve extracted from the AFM measurement is larger than the energy dissipation actually experienced by the molecule (Fig. 2b). To obtain a more accurate estimation of the energy dissipation by the molecules we propose an algorithm to correct the energy dissipation values obtained from integrating the area under the measured SMFS curve.

### Corrections to the energy dissipation

**Figure 3:**
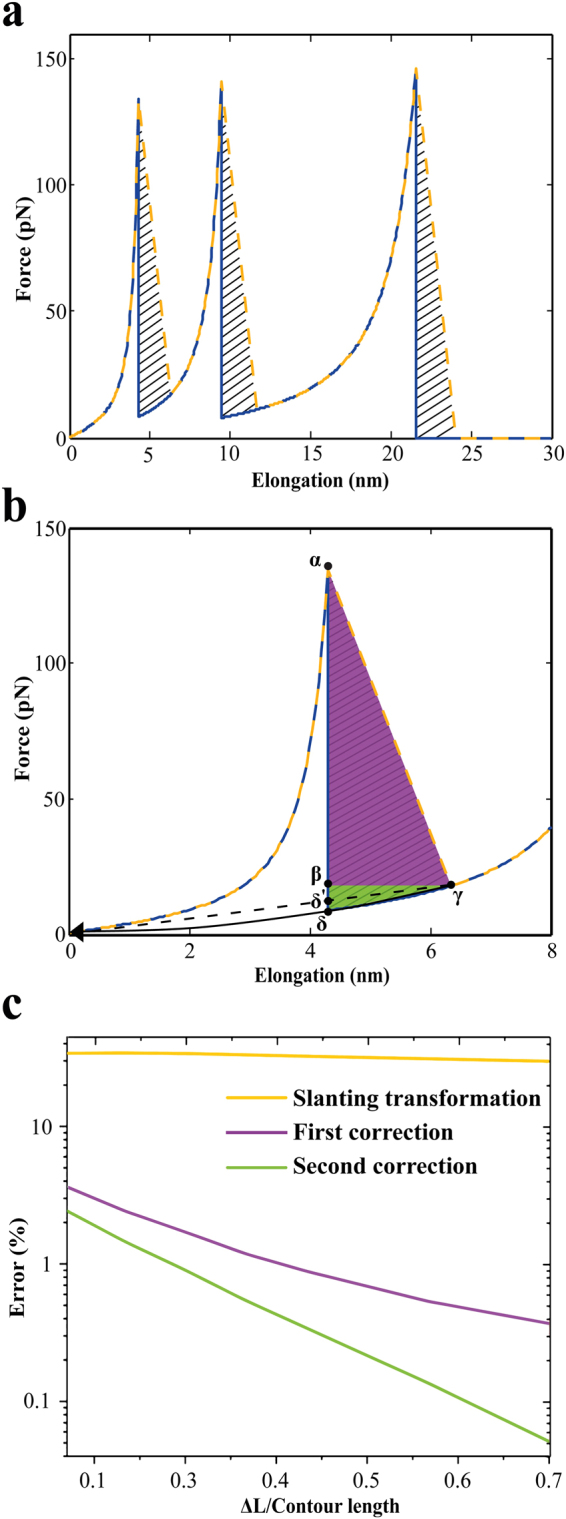
Consecutive steps for calculating the correct dissipated energy value by eliminating the artefact arising from the finite stiffness of the cantilever. (**a**) Shows a simulated pulling curve with three peaks (corresponding to three rupture events) using worm-like chain (WLC) model. The dark blue continuous line represents the ideal WLC curve and the orange dashed line represents the real case scenario (considering the finite stiffness of the cantilever). The shaded regions marked with black lines for all the three peaks indicate the areas that need to be eliminated to correct for the overestimation in the energy dissipation. (**b**) Shows the magnified version of the first peak shown in (**a**). By calculating the area of the two right-angled triangles αβγ (purple triangle) and βγδ (green triangle), we can determine the excess energy that has to be subtracted to get the correct value of the dissipated energy. (**c**) Magnitude of the overestimation in the dissipated energy values (as percentage error) between the exact WLC curve and the reconstructed curve, before and after incorporating the two correction factors to calculate the correct energy dissipation, as a function of the ratio of hidden length and contour length. The orange curve shows the plot of dissipated energy difference as percentage error between the exact WLC curve and the reconstructed curve before incorporating any correction factors. The purple curve represents the plot of dissipated energy difference as percentage error between the exact WLC curve and the reconstructed curve after eliminating the area of the triangle αβγ. Similarly, the green curve shows the difference in dissipated energy of the exact WLC curve and the reconstructed curve after eliminating the areas of the two triangles αβγ and βγδ.

Since no actual information is available for the force exerted on the molecules during the snap back, it is not possible to calculate the accurate energy dissipation during the whole pulling cycle (see the shaded regions marked with black lines in Fig. 3a. The best we can do is to interpolate this region. Ideally this would be done with one of the models used for molecular extension (such as the WLC model). However, since in real experimental data the signal to noise ratio is often not good enough, or the distance between ruptures is too short to fit a WLC model, we approximate the area of over estimation using two easily calculable triangles.

Figure 3a shows a simulated pulling curve with three peaks (corresponding to three rupture events) calculated using the WLC model. The dark blue continuous line represents the ideal WLC curve and the orange dashed line represents the “as measured” scenario (considering the finite stiffness of the cantilever). The shaded regions marked with black lines for all the three peaks indicate the areas that need to be eliminated to correct for the overestimation in the energy dissipation. Figure 3b shows the magnified version of the first peak of Fig. 3a.

To determine the excess energy, we have to calculate the area of the region marked by α, γ and δ as shown in Fig. 3b. To do so, we have considered two right-angled triangles - denoted by αβγ (purple triangle) and βγδ (green triangle), and calculated the areas of these two triangles, denoted by A_1_ and A_2_ respectively. To achieve the final corrected energy dissipation value, the areas of these two triangles are subtracted from the integrated area under the force curves. For the remainder of this manuscript we call the correction of the purple triangle the “first correction” and the correction of the green triangle the “second correction”.

The algorithm is implemented in custom software written in Matlab (see supplemental information for details). The program is designed to automate the processing of SMFS curves taken using AFM, and calculates the area of the two triangles to correct for the overestimated energy values calculated from force spectroscopy curves. In short, the algorithm first performs a baseline correction for the pulling curve. Then it applies the slanting transformation based on the known deflection sensitivity to transform the force-displacement curves into force-elongation curves. The rupture peaks are then identified using differentiation of the filtered force curves.

To calculate the area for the “first correction”, a right-angled triangle (purple triangle in Fig. 3b is created by considering the last data point before the rupture (α) and the first data point after the rupture (γ). The other right-angled triangle (green triangle shown in Fig. 3b is created by performing the quadratic regression between origin and the point γ and finally, approximating the length of the curve between the points δ and γ as a straight line. Alternatively, this right-angled triangle can also be created by extrapolating the first point after the fracture (γ) to the point of zero extension, that is, by performing linear regression between origin and the point γ instead of quadratic regression (as shown in Fig. 3b using a dashed black line). The area of this second right-angled triangle (green triangle shown in Fig. 3b) provides the “second correction” factor mentioned above. The areas of these triangles are then subtracted from the dissipated energy as calculated by numerically integrating the area under the force-elongation curve. As evident from Fig. 3b, quadratic regression provides more accurate value for the “second correction” factor compared to linear regression, hence quadratic regression method is used for all the data shown in this paper unless stated otherwise.

### Estimation of residual errors in the calculated energy dissipation

The amount of over estimation of the energy dissipation when calculated only by integrating the area under the curve depends on the exact configuration of the molecules in the network and the amount of hidden length released by each rupture. To characterize how extensive the over estimation is we have performed simulated experiments with different amounts of hidden length (ΔL) and calculated the energy dissipation from the ideal WLC curves (as shown in Fig. 2b), and from integrating the curve as would be measured by the AFM SMFS experiment as shown in Fig. 2e. The relative difference between these two energies is shown as the orange curve in Fig. 3c.

The purple curve represents the remaining error between the ideal energy dissipation value and the calculated value after applying the “first correction”. Similarly, the green curve shows the difference in dissipated energy as percentage error after applying both the “first” and “second correction”. For all the three curves the relative error decreases as the hidden length of the molecule increases. Maximum error calculated for the slanting transformed curve (orange curve in Fig. 3c) is 34.13%, whereas it decreases to 3.69% after incorporating the first correction factor (purple curve in Fig. 3c) and to 2.49% after second correction (green curve in Fig. 3c) for 30 nm contour length. The estimated values of the excess dissipated energy depends strongly on the model assumptions for this work.

### Experimental results

**Figure 4:**
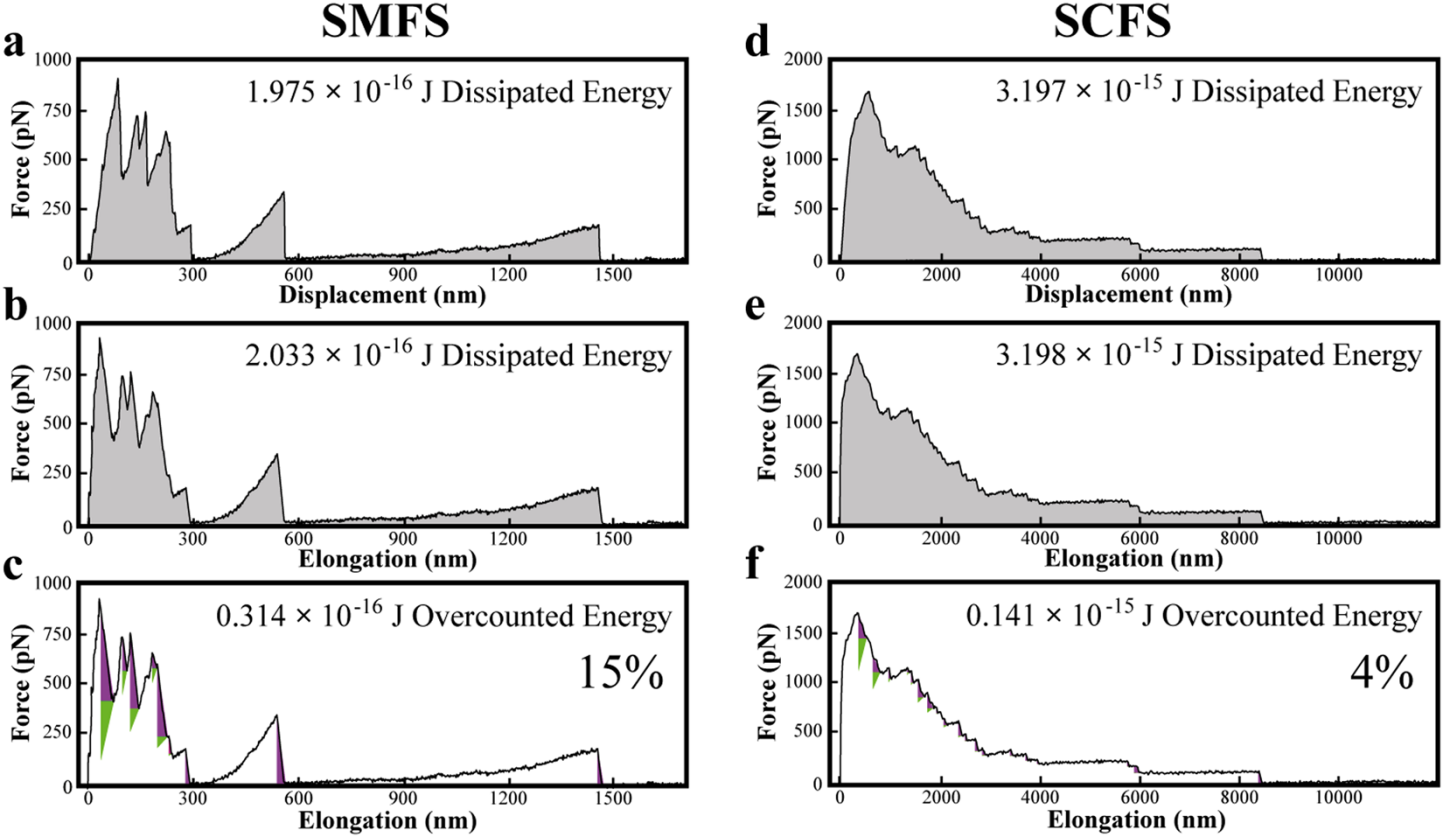
Significance of the correction factors for calculating energy dissipation from a typical SMFS curve on a molecular network and a SCFS curve taken on a mammalian HeLa cell. The pulling curve (SMFS) has been taken on Osteopontin deposited on polished hydroxyapatite surface in the presence of Na buffer. (**a**) Shows the force vs. displacement curve (without slanting transformation) as measured from the SMFS data. The shaded grey area between the approach curve and retraction curve represents the total energy dissipation during the pulling event. In this case the calculated value for the energy dissipation is 1.975 × 10^−16^ J. (**b**) Shows the same pulling curve after applying the slanting transformation. The dissipated energy value changes to 2.033 × 10^−16^ J. (**c**) shows the same slanting transformed pulling curve as in (**b**), except that the two correction factors have also been applied while calculating the actual dissipated energy value. The overestimated areas are marked by the two triangles (purple and green) at each rupture point of the retraction curve. After applying this correction, the dissipated energy value decreases by 0.314 × 10^−16^ J, which corresponds to 15.44%. (**d**) Shows the force vs. displacement curve (without slanting transformation) as measured from the SCFS data. The shaded grey area between the approach curve and retraction curve represents the total energy dissipation during the pulling event. In this case the calculated value for the energy dissipation is 3.197 × 10^−15^ J. (**e**) Shows the same pulling curve after applying the slanting transformation. The dissipated energy value changes to 3.198 × 10^−15^ J. (**f**) shows the same slanting transformed pulling curve as in (**b**), except that the two correction factors have also Been applied while calculating the actual dissipated energy value. The overestimated areas are marked by the two triangles (purple and green) at each rupture point of the retraction curve. After applying this correction the dissipated energy value decreases by 0.141 × 10^−15^ J, which corresponds to 4.41%.

Figure 4 demonstrate the significance of the abovementioned correction factors, described in the previous sections, for calculating the energy dissipation from a typical SMFS curve and a single cell force spectroscopy (SCFS) curve respectively. The SMFS curve has been taken on Osteopontin (one of the most abundant non-collagenous proteins present in bone) deposited on polished hydroxyapatite surface in presence of *Na buffer* (see methods section for buffer description). Figure 4a shows the force vs. displacement curve (without slanting transformation). The shaded grey area between the approach curve and retraction curve represents the total energy dissipation during the pulling event.

In this case the calculated value for the energy dissipation is 1.975 × 10^−16^ J. Figure 4b shows the same pulling curve after applying the slanting transformation. After slanting transformation, the calculated energy value changes to 2.033 × 10^−16^ J, which corresponds to 2.85%. Figure 4c depicts the same curve as in Fig. 4b, except that the two corrections have been applied while calculating the actual dissipated energy value. The overestimated areas are marked by the two triangles (purple and green) at each rupture point of the retraction curve. After applying these corrections the dissipated energy value decreases by 0.314 × 10^−16^ J compared to only the slanting transformed case (shown in Fig. 4b), which corresponds to 15.44%.

Force distance curves of single cell force spectroscopy (SCFS) often show a similarly complex behavior as those for molecular networks. We evaluated SCFS curves that have been taken on mammalian HeLa cell. Figure 4d shows the force vs. displacement curve (without slanting transformation). The shaded grey area between the approach curve and retraction curve represents the total energy dissipation during the pulling event. In this case the calculated value for the energy dissipation is 3.197 × 10^−15^ J. Figure 4e shows the same pulling curve after applying the slanting transformation. After slanting transformation, the calculated energy value changes to 3.198 × 10^−15^ J, which corresponds to 0.01%. Figure 4f depicts the same curve as in Fig. 4e, except that the two corrections have been applied while calculating the actual dissipated energy value. The overestimated areas are marked by the two triangles (purple and green) at each rupture point of the retraction curve. After applying these corrections the dissipated energy value decreases by 0.141 × 10^−15^ J compared to only the slanting transformed case (shown in Fig. 4e), which corresponds to 4.41%.

## Discussion

**Figure 5:**
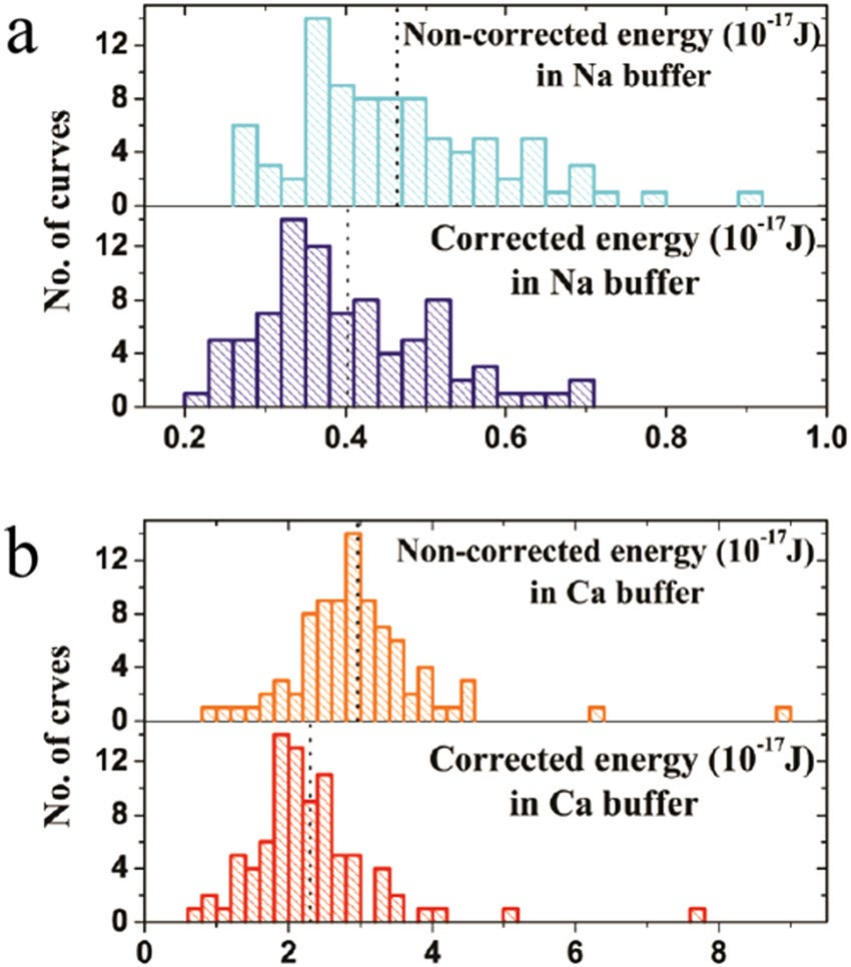
Shows the statistical distribution of the total energy dissipation of several pulls for Na buffer (**a**) without correction (upper panel), with correction (lower panel) and similarly for Ca buffer (**b**) without correction (upper panel) and with correction (lower panel). The mean values for all four cases are shown as black dashed lines in both the panels of (a) and (b). It is evident that the statistical distributions of non-corrected and corrected energy dissipation values differ significantly for both Na and Ca buffer solutions.

As shown in the previous section, the error in the dissipated energy that is introduced by transforming the force vs. displacement curves into force vs. elongation (separation) curves can be a significant fraction of the total energy dissipation. To show how significant this error can be when studying biological networks, we use the interaction between Osteopontin and the surface of mica, a model system for the interaction of bone organic and inorganic matrix^22–24^. We performed pulling experiments in two different buffer solutions Na buffer (control buffer solution) and Ca buffer, respectively, to investigate the effect of the divalent ions on the interaction between mica and Osteopontin^22,24^. Figure 5 shows the statistical distribution of the total energy dissipation over several pulls for Na buffer without correction (upper panel of Fig. 5a), with correction (lower panel of Fig. 5a) and similarly for Ca buffer without correction (upper panel of Fig. 5b) and with correction (lower panel of Fig. 5b), respectively. Large amounts of energy are dissipated in these pulls, and the total energy dissipation increases with presence of Ca^2+^ ions as has been reported in^22,24^. It is evident from Fig. 5 that the statistical distributions of non-corrected and corrected energy dissipation values differ significantly for both Na buffer and Ca buffer solutions. The mean values for non-corrected and corrected energy dissipation are (0.46 ± 0.13) × 10^−17^ J and (0.40 ± 0.11) × 10^−17^ J, respectively for Na buffer. For Ca buffer these values are (2.96 ± 1.03) × 10^−17^ J and (2.30 ± 0.93) × 10^−17^ J, respectively. The mean values are shown as black dashed lines in both the panels of Fig. 5a and b.

It is important to mention here that SCFS curves are more complex than SMFS curves due to the combined effect of different molecules present in the cells and the mechanical properties of the cell membrane itself^26^. The more complex the interaction between the tip and the sample becomes, the more difficult it is to distinguish between clear artefacts due to the tip snap back and real energy dissipation mechanisms. Nevertheless, the artefact at the end of any broken connection leading to an uncontrolled snap back of the cantilever will cause an over estimate, irrespective of the fact that the connection first was a membrane tether or a single molecule. The difference between the two manifests itself in how abrupt the transition on the peak is and how fast the relaxation of the cantilever deflection. Viscous effects will slow down the motion of the cantilever, and it will therefore not result in an instantaneous change in the cantilever position. In our algorithm, we see this difference by looking at the first derivative of the force curve. An abrupt transition will cause a high value, indicating a clean break. A low value indicates a gradual drop indicating viscous interactions. The level of the threshold in the peak finding routine can be adjusted based on a-priori knowledge and experience of the experimenter.

## Summary and Outlook

Calculating the energy dissipation directly from the slanting transformed pulling curve can lead to a significant over-estimation of the dissipated energy during a pulling experiment on molecular networks or during cell-adhesion measurements. In the example we show in this paper, the relative error is 13.04% for experiments performed in Na-buffer, and 22.30% for experiments performed in Ca-buffer. These are substantial miss-calculations that could mask physiologically important differences during such experiments. While extracting the exact energy dissipation is difficult, implementing a simple subtraction of two right-angled triangles reduces the remaining error to a few percent. How well this simple correction estimates the true energy dissipation depends on the exact shape of the pulling curves. It should be evaluated for each different type of experiment if these corrections are sufficient or if additional corrections need to be implemented. The algorithm presented in this manuscript can also serve as a base platform for more specialized corrections should they be required. However, already applying the simple two-triangle approximation significantly reduces the error made in the calculated energy dissipation for many force spectroscopy experiments.

## Methods

### Osteopontin

Recombinant human Osteopontin (OPN) was purchased from Sigma Aldrich (SRP3131-50UG) and dissolved in MILLI-Q (Millipore, Billerica, MA, USA) water to a concentration of 0.2 μg/μL. Aliquots of the prepared solution were stored at −20 °C in micro centrifuge tubes. Before each experiment, 4 μL of the solution was deposited onto a freshly cleaved mica surface and allowed to dry. The sample was subsequently placed on the scanner of a Multimode IIIa AFM system with Picoforce extension (Bruker Nano: Santa Barbara, CA, USA). Samples were then rehydrated in the Na^+^ buffer solution inside the fluid cell.

### Buffer solutions

We used the following buffer solutions for this study.

1. Na buffer solution - 150 mM NaCl, 10 mM HEPES, pH 7.4, was used as a control buffer solution.
2. Ca buffer solution - 40 mM CaCl_2_, 110 mM NaCl, 10 mM HEPES, pH 7.4.

### Atomic force spectroscopy measurements

The AFM force spectroscopy was done using a Multimode IIIa AFM system with PicoForce (Bruker Nano: Santa Barbara, CA, USA). We used Hydra-All-G (Silicon Nitride cantilevers, gold coated on the reflex side) cantilever (B) from Applied NanoStructutres, Inc. (CA, USA) with a nominal spring constant of 0.045 N/m (manufacture’s value). For proper calibration, the spring constant of the cantilever was determined using the thermal tune method.

The pulls were performed in a 5 × 5 grid with a spacing of 1 µm. For each pull, we followed this procedure: the cantilever was pressed onto the surface with 500 pN force (relative trigger mode) for 3 seconds and then retracted at a speed of 190 nm/s. After each grid was completed, an exchange of the buffer solution (200 µl) was made. For each buffer solution, the grid was repeated 4 times before flushing with the next buffer solution. A total of 100 pulls were therefore recorded per buffer solution. The energy dissipation was calculated from each individual pulling curve using the abovementioned MATLAB data analysis program.

### Mammalian cell culture conditions

HeLa cells (generously provided by the Laboratory of nanoscale biology group, École Polytechnique Fédérale de Lausanne), were grown in high glucose DMEM (Gibco) supplemented with 10% fetal bovine serum, 4 mML glutamine, 1 mM sodium pyruvate (Sigma), in 5% CO_2_ at 37 °C. Prior to the adhesion force measurements, the cells were washed with a filtered CO_2_-independent medium that is identical with the cell culture medium above but supplemented with 10 mM HEPES. The cell concentration was adjusted to obtain sufficient numbers of individual isolated cells on the desired substrate at the start of the experiment.

### Force spectroscopy measurements on cells

The cell adhesion forces were measured on mammalian HeLa cells using a Multimode IIIa AFM system with PicoForce (Bruker Nano: Santa Barbara, CA, USA). For the adhesion measurements we used tip-less silicon nitride cantilevers (NP-O10, Bruker), functionalized overnight with 2 mg/ml concanavalin A. The spring constant of the cantilever was calibrated using Thermal tune method before performing the experiments (0.068 N/m). The deflection sensitivity of the cantilever (43 nm/V) was also measured before starting the experiments by acquiring force curves on the cell free areas of the glass bottom petri dishes. For measuring the cell adhesion force, the cell was approached towards the surface coated overnight with 10 µg/mL fibronectin with a set force of 1 nN and a speed rate of 10 μm/s. After a contact time of 5 s between the cell and the fibronectin, the sample was retracted 18 μm with a speed rate of 5um/s. The deflection signal of the probe was recorded as a function of retraction until the cell was removed from the surface.

### Data availability

All data generated or analyzed during this study are included in this published article (and its Supplementary Information files).

## Electronic supplementary material

Supplementary information

Example 1

Example 2

Example 3

Matlab code

Matlab code

Matlab code

Matlab code

Matlab code

Matlab code

Matlab code

Matlab code

Matlab code

Matlab code

Matlab code

Matlab code

Matlab code

Matlab code
