# Supplementary material for: Reducing uncertainties in energy dissipation measurements in atomic force spectroscopy of molecular networks and cell-adhesion studies
Source: Sci Rep. 2018 Jun 20;8:9390. doi: 10.1038/s41598-018-26979-0 (PMC6010446; doi:10.1038/s41598-018-26979-0)
Supplement: Supplementary file 1 — Supplementary information [file 41598_2018_26979_MOESM1_ESM.docx]

**SUPPLEMENTARY INFORMATION**

**Reducing uncertainties in energy dissipation measurements in atomic force spectroscopy of molecular networks and cell-adhesion studies**

Soma Biswas^1*^, Samuel Leitao^1*^, Quentin Theillaud^1^, Blake W. Erickson^1^, and Georg E. Fantner^1^

^1^Laboratory for Bio- and Nano-Instrumentation, École Polytechnique Fédérale de Lausanne, Batiment BM 3109 Station 17, 1015 Lausanne, Switzerland.

^*^These authors contributed equally to this work.

Correspondence and requests for materials should be addressed to G.E.F. (email: georg.fantner@epfl.ch).

**MATLAB program**

This section describes the sequential order of data processing executed in the MATLAB program. It is designed to automate the processing of the SMFS curves. The schematic of the data processing flow is represented in Figure S1. The first step of the process flow is to specify the folder containing the data set and the file name. Start and end numbers define the first and the last file to work on. This allows analyzing a small subset of the data or the whole data set. This option is particularly useful if the user knows that a portion of the measurements is not ideal or if there is an anomaly in some part of the experiment. The parameters that control the analysis of the data have to be tuned very carefully for each data set by observing first few curves before analyzing the whole data set. Any rupture peak that is considered for analysis has to be bigger than the noise threshold (multiple of the RMS noise in the baseline) defined in the program. After importing the data, approach and retraction data points are processed and then the baseline is set to zero. The slope of the force curve is fitted with polynomial to find the intersection with the baseline. In the next step, piezo displacement is transformed to actual tip-sample separation by subtracting the cantilever deflection from the z-piezo position. Finally, the program plots the corrected approach and retraction force-displacement curves.


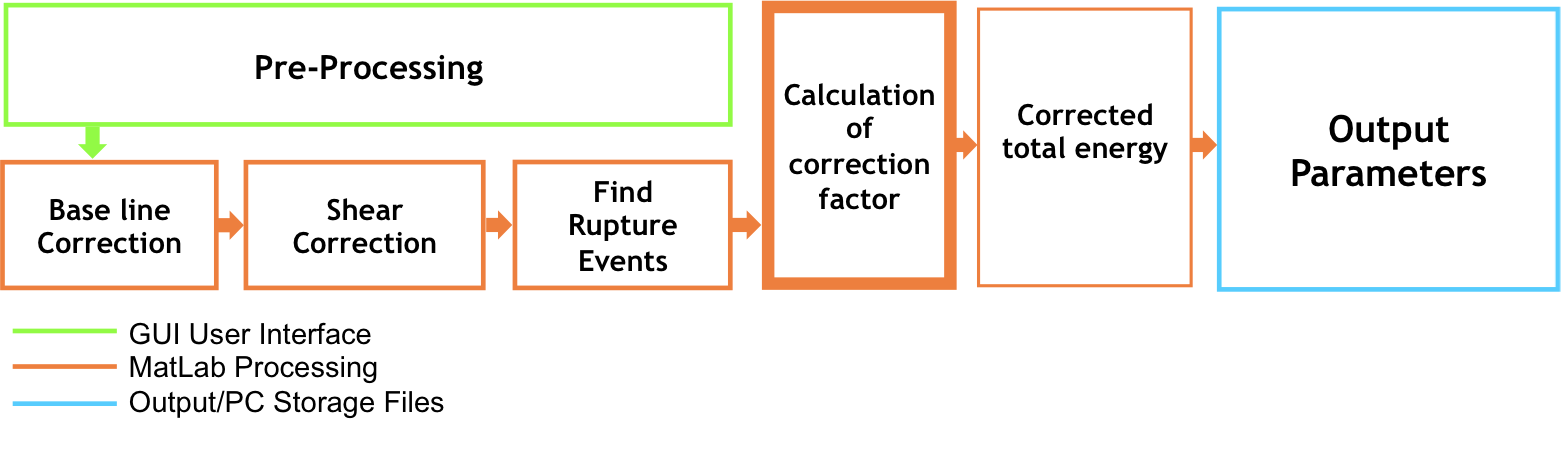


Figure S1. The block diagram represents the data processing flow using the MATLAB based algorithm. The first step of this program is to load the pulling curves and extract the parameters followed by data processing. The data processing is initiated by base line and slanting correction, and then the rupture events are identified. Finally, overestimation of the energy is calculated and the corrected total energy has been obtained. At the end, output parameters, such as, corrected and non-corrected total energy, maximum pull force and total pull length are obtained.

To find the peaks corresponding to the rupture events, a Savitzky-Golay filter has been applied to the derivated data and the peaks are identified by recognizing the point, where the derivative changes sign. The algorithm finds out the starting and the ending point of the rupture events and also eliminates the events smaller than noise. It also provides an option for the users to select manually the peaks that they consider as rupture events. After selecting the rupture events, the energy corrections to the determined peaks have been calculated. Finally, maximum force, pull length and total dissipated energy (including and excluding the correction factors) values are calculated for each SMFS curve as shown in Figure S2.


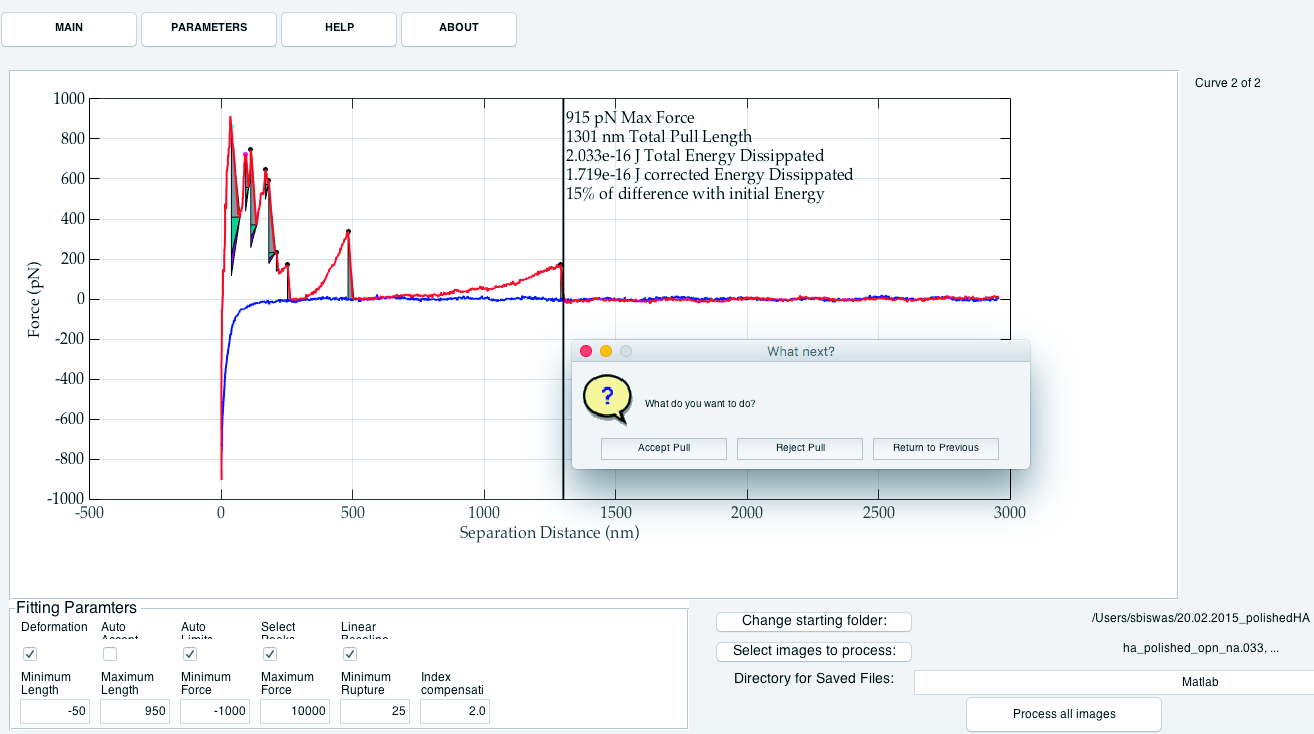


**Figure S2.** It shows different options (such as, base line correction, slanting correction, peak selection) available to the users in graphical user interface (GUI) of the MATLAB program for analyzing the force spectroscopy data. The approach and retraction curves are shown using blue and red colors respectively. The black vertical line represents the total pull length.
